# Ultrasonic Transformation of Antibiotic Molecules into a Selective Chemotherapeutic Nanodrug

**DOI:** 10.3390/molecules28134927

**Published:** 2023-06-22

**Authors:** Haiyan Zhu, Sukhvir Kaur Bhangu, Muthupandian Ashokkumar, Francesca Cavalieri

**Affiliations:** 1 School of Chemistry, The University of Melbourne, Parkville, Melbourne, VIC 3010, Australia; haiyan.zhu@rmit.edu.au; 2 School of Science, RMIT University, Melbourne, VIC 3000, Australia; roop.bhangu@csiro.au; 3 Department of Chemical Sciences and Technologies, University of Rome Tor Vergata, Via della Ricerca Scientifica 1, 00133 Rome, Italy

**Keywords:** ultrasound, doxycycline, nanoparticles, anticancer, intracellular trafficking

## Abstract

Ultrasound-based engineering of carrier-free nanodrugs by supramolecular self-assembly has recently emerged as an innovative and environmentally friendly synthetic approach. By applying high-frequency sound waves (490 kHz) in aqueous solutions, the transformation of small chemotherapeutic and antibiotic drug molecules into carrier-free nanodrugs with anticancer and antimicrobial activities was recently achieved. The transformation of the antibiotic drug molecules, i.e., doxycycline, into stable nanodrugs (~130 nm) with selective anticancer activity was achieved without requiring organic solvents, chemical agents, or surfactants. The obtained nanodrug exhibited reactive oxygen species (ROS)-mediated cytotoxicity on human breast cancer (MDA-MB 231 cells) but a negligible antiproliferative effect on healthy fibroblast cells. Imaging by super-resolution microscopy (STORM) provided insights into the intracellular trafficking and endosomal escape of the nanodrugs. Overall, these findings suggest that small antibiotic drugs can be transformed into chemotherapeutic nanodrugs with high selectivity against cancer cells.

## 1. Introduction

Breast cancer is one of the most common cancers, accounting for nearly 30% of all cancer diagnoses among women [1,2]. It has been found that various factors, including the inheritance of mutations in the genes of BRCA2, BRCA1, and CHEK2, exposure to female hormones, and chronic inflammation, are the main causes of breast cancer [3,4,5]. The common treatment for breast cancer involves surgery, radiation therapy, chemotherapy, hormone therapy, and targeted therapy. Recently, novel anticancer agents have been identified among numerous antibiotics [6,7,8,9]. It has been found that antibiotics (actinomycin, monensin, ivermectin, doxycycline, and salinomycin) could also have synergistic anti-tumor effects by inhibiting anti-apoptotic proteins and proteins involved in angiogenesis and migration of tumor cells [8,9,10].

Doxycycline is a broad-spectrum antibiotic with tetracene ring structures that belongs to the tetracycline class of antibiotics. It was first discovered in the 1960s and is derived from the natural product oxytetracycline, which was isolated from *Streptomyces aureofaciens* [11,12]. Doxycycline has since become a widely used antibiotic due to its efficacy against a broad range of bacteria, including both *Gram*-positive and *Gram*-negative bacteria [13,14]. The antibiotic mechanism of doxycycline involves inhibiting bacterial protein synthesis by binding to the 30S ribosomal subunit and then preventing the binding of aminoacyl-tRNA, thereby disrupting the supply of amino acids for protein biosynthesis [15]. Moreover, doxycycline, as a cytostatic antibiotic, can block the synthesis of mitochondrial proteins, resulting in anti-proliferating properties in cells [8]. 

One of the biggest challenges for drug delivery in cancer treatment is non-selectivity, which means drug molecules not only kill or slow the growth of cancer cells but also affect nearby healthy cells, leading to several side effects such as nausea, hair loss, fatigue, and pain. Nanoparticle-mediated delivery of drugs is a promising approach for cancer treatment that involves the use of nanomaterials to deliver drugs directly to cancer cells while reducing damage to healthy cells. Doxycycline conjugated with or loaded into polymeric, metallic, or liposome nanoparticles has shown considerable antitumor effects on various types of cancer cells [7,12,16,17,18]. Copper nanoparticles conjugated with doxycycline showed excellent antitumor effects on HeLa and HepG2 cells in a synergistic manner [18]. Polymeric doxycycline nanoparticles formulated by the nanoprecipitation method using hydroxypropyl methylcellulose as a polymer showed greater antitumor potential against Ehrlich carcinoma [19]. Although the nanoparticles loaded with antibiotics have shown anticancer efficacy, collateral safety issues, such as cytotoxicity and the proinflammatory response exerted by the encapsulating nanomaterials, remain unsolved. In this case, a better way to design nanodrug platforms is to fabricate nanoparticles composed entirely of the active agents.

The ultrasonic technique has recently emerged as a new and environmentally friendly method for synthesizing nanomaterials in various fields, such as organic, inorganic, and biomedical applications [20,21,22,23,24,25,26]. The interaction between ultrasonic waves and dissolved gas bubbles in a liquid leads to acoustic cavitation processes. Extreme temperatures are generated during the near-adiabatic collapse of cavitation bubbles. In an aqueous medium, water molecules present within the collapsing cavitation bubbles are decomposed to generate highly reactive radicals. We recently reported that the transformation of the non-selective anthracycline doxorubicin into a tumor-selective nanodrug could be achieved by the reaction between OH radicals and biomolecules on the surface of cavitation bubbles generated using high-frequency ultrasound [27]. We have also shown that the antibiotic doxycycline is responsive to high-frequency ultrasound (490 kHz), and a carrier-free nano-antibiotic with good antimicrobial and antioxidant activity was obtained using this innovative ultrasonic strategy [28].

In this study, we sought to investigate whether the ultrasonically modified doxycycline molecules can exert a selective anticancer activity against breast cancer cells (MDA-MB-231). We investigated the anticancer properties of DoxyNPs against breast cancer cells by evaluating the particles’ biostability, cell association, cytotoxicity, and endosomal escape performance. The sono-assembled DoxyNPs showed an anti-proliferation effect on MDA-MB-231 due to the mediated generation of reactive oxygen species (ROS), with a negligible effect on healthy fibroblast cells. This study paves the way for a possible future application of sono-assembled DoxyNPs as anti-tumor drugs to improve efficacy in cancer treatment.

## 2. Results and Discussion

**Figure 1:**
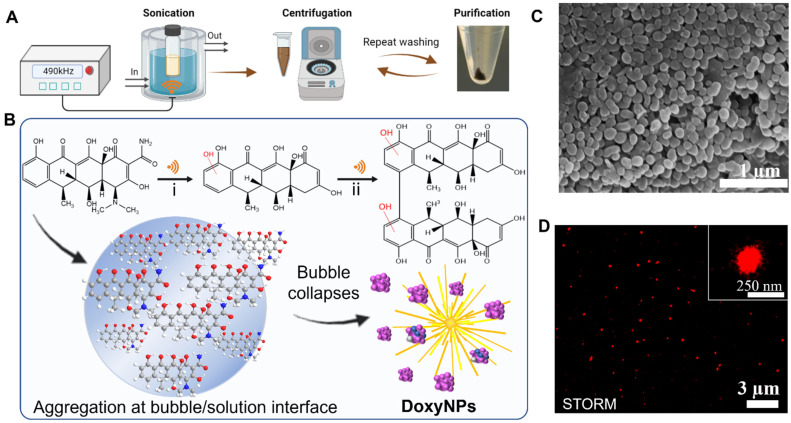
Sonochemically synthesized doxycycline nanoparticles (DoxyNPs). (**A**) Schematic diagrams show the procedure of forming sono-assembled DoxyNps using a 490 kHz setup at 20 W cm^−3^ for sonicating for 1 h, and with the symbol “
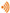
” representing ultrasound; (**B**) Schematic of the ultrasound-induced chemical modification of doxycycline and self-assembly into nanodrugs during cavitation bubble collapse; (**C**) SEM images of obtained DoxyNPs upon 1 h sonication using 490 kHz ultrasound at 20 W cm^−3^; (**D**) Representative STORM imaging of DoxyNps labeled with AF 647 NHS-Ester dye; inset showed a magnified view of a single DoxyNPs.

Doxycycline nanoparticles (DoxyNPs) were obtained by sonicating 1 mg/mL doxycycline (Doxy) aqueous solution for 1 h using a 490 kHz ultrasound setup at 14.4 W cm^−3^ (yield~78%). The obtained DoxyNPs were purified by repeated washing and centrifugation steps (Figure 1A). The mechanism of DoxyNP formation was previously elucidated [28]. Briefly, the OH radicals generated by the acoustic cavitation induce structural changes in the doxycycline aromatic molecules. The radical-mediated oxidation of doxycycline results in the cleavage of amine/amide moieties and hydroxylation and dimerization processes (Figure 1B). The obtained amphiphilic molecules ultimately self-assemble into nanoparticles during cavitation bubble collapse due to intermolecular interactions, i.e., H-bonding and *π*–*π* interactions [28].

The average sizes of the obtained particles measured from SEM images are around 132 ± 26 nm in the dry state (Figure 1C). The sono-synthesized DoxyNPs were labeled with AF 647 dye and visualized with nanoresolution by stochastic optical reconstruction microscopy (STORM) (Figure 1D) in the hydrated state. The average size of DoxyNPs in the hydrated state was around 213 ± 35 nm, as determined by STORM, and the zeta potential of DoxyNPs was −32 mV.

**Figure 2:**
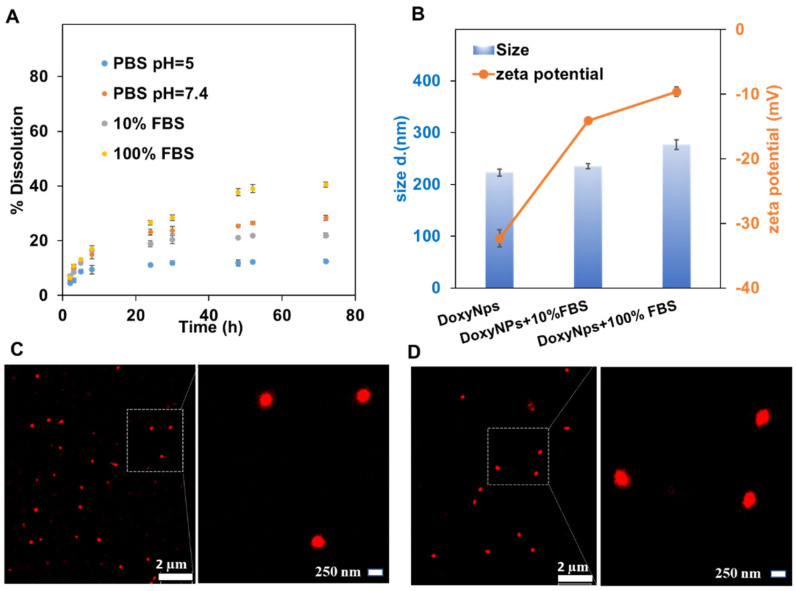
Bio-stability and kinetic release of DoxyNPs. (**A**) Release kinetics of DoxyNPs suspensions under various simulated physiological conditions, including 100 mM PBS at pH 5 and pH 7.4, cell culture medium (DMEM) containing 10% FBS and 100% FBS medium, respectively. The dissolution percentage was calculated by measuring the intensity of fluorescence emission (excited at 360 nm) of the collected supernatant after centrifugation of DoxyNP suspensions. FV (**B**) Size and zeta potential of DoxyNPs incubated with 10% FBS and 100% FBS phosphate-buffered saline solutions. (**C**) STORM images of the DoxyNPs (labeled with Alexa fluor 647 NHS-Ester) acquired after 3 h incubation with 10% FBS (**C**) in phosphate-buffered saline solution and 100% (**D**) FBS medium, respectively.

Tan et al. first reported that doxycycline can induce mitochondrial dysfunction and oxidative damage in cancer cells through intracellular ROS generation [29]. We hypothesized that DoxyNPs may also possess anti-cancer activity. First, the stability of DoxyNPs in solutions mimicking physiological conditions was investigated by monitoring the dissolution of nanoparticles as a function of time. A marginal dissolution of DoxyNPs was observed in phosphate buffer saline (PBS) at pH 5 (2%), pH 7.4 (5%) in 10% FBS (3%), and 100% FBS (14%), after 24 h of incubation (Figure 2A). At physiological pH conditions, the deprotonation of hydroxyl (-OH) groups of DoxyNPs bearing aromatic rings probably resulted in the slow disassembling of DoxyNPs due to the repulsive electrostatic interactions between the negatively charged drug molecules (Figure 2B).

It should be noted that the presence of serum proteins (10% and 100% FBS) did not cause significant aggregation or dissolution of DoxyNPs (Figure S1), as shown by STORM imaging of single nanoparticles (Figure 2C,D). The zeta potential of DoxyNPs decreased from −32 mV to −10 mV after incubation with FBS, indicating the effective formation of the protein corona around the DoxyNPs (Figure 2B). The adsorption of proteins onto DoxyNPs is likely mediated by hydrophobic or electrostatic interactions. Overall, these results demonstrated that DoxyNPs are stable under biological conditions and capable of releasing the drug molecules gradually over a long period of time.

The biological activity of DoxyNPs is primarily dependent on their cellular uptake. The efficiency of internalization of DoxyNPs in MDA-MB 231 cells was evaluated by flow cytometry studies. DoxyNPs were coated with fluorescently labeled transferrin by physical adsorption since the transferrin receptor is an attractive molecule for the targeted delivery of nanoparticles to cancer cells. It is upregulated on the surface of various cancer cells and enables the efficient internalization of transferrin-conjugated nanoparticles [30,31,32]. 

**Figure 3:**
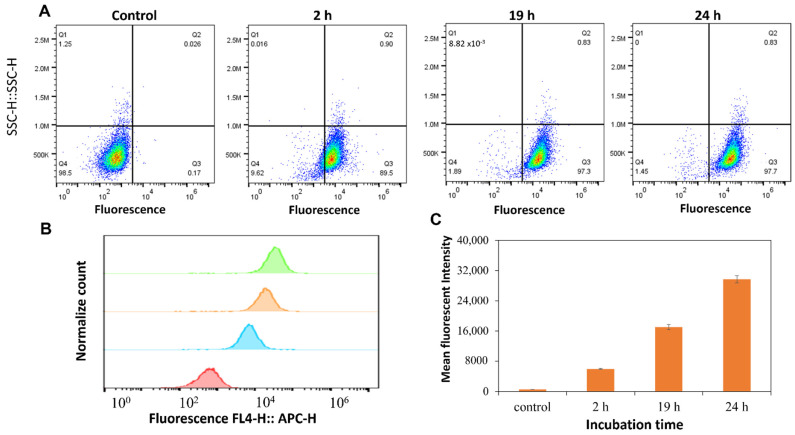
Association of transferrin-coated DoxyNps labeled with AF 647 and MDA-MB-231 (breast cancer cells) up to 24 h. (**A**) Flow cytometry pseudocolor plots of DoxyNP’s association with MDA-MB-231 cells via gating strategy (cell debris excluded). Background fluorescence from untreated cells was measured as negative controls to define positivity in each channel. Q3 represents the association and uptake of particles by cancer cells. (**B**) Histogram of DoxyNP’s association with MDA-MB-231 after 2 h, 19 h, and 24 h; (**C**) Mean fluorescence of untreated MDA-MB-231 cells and after 2 h, 19 h, and 24 h treated with DoxyNPs.

By using the flow cytometry gating strategy, the effect of cell intrinsic fluorescence was eliminated (0.17% from Q3) (Figure 3A). The association between particles and cells increased from 0.17% to 95.8% (Figure 3A) with increasing incubation times up to 24 h. In the first two hours, MDA-MB 231 cells had already taken up more than 60% of the particles. Figure 3B showed that the fluorescence intensity in the FL4 channel (647 nm) shifted to higher intensity values compared to that of the control samples (Figure 3C). These results suggested that the DoxyNP cells are progressively internalized inside the cells over time. 

**Figure 4:**
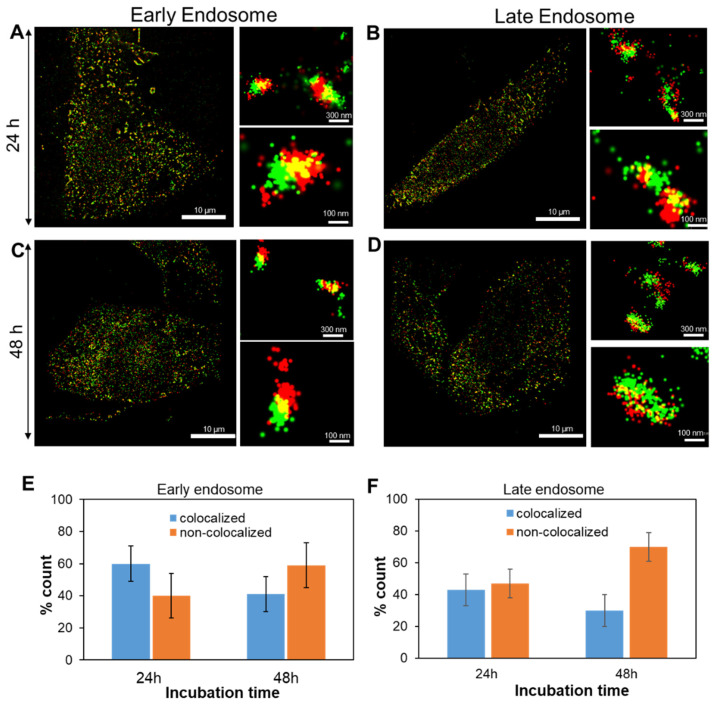
Intracellular trafficking study of DoxyNPs in MDA-MB-231 cells by stochastic optical reconstruction microscopy (STORM). Representative multicolor STORM images of the MDA-MB-231 cells exposed for 24 h to DoxyNPs (red signal) and stained for early (**A**) and late endosomes (**B**) (green signal). (**C**,**D**) represent 48 h for early and late endosomes, respectively. The high-magnification images (scale bars of 300 nm and 100 nm) provide more details of the interaction between intracellular vesicles (red) and DoxyNPs (green). (**E**,**F**) Statistical analysis of colocalized and non-colocalized DoxyNPs with early and late endosomes as a function of incubation time.

To investigate the intracellular trafficking and subcellular localization of DoxyNps, we performed super-resolution imaging (STORM) studies. For STORM imaging, the fluorescent transferrin-coated DoxyNPs were prepared by the dual labeling method (AF 556/647), as described in Section 3.7. MDA-MB-231 cells were dual-labeled by a secondary antibody (AF 488/647) for early endosomes and late endosomes. The images taken by confocal microscopy confirmed the cellular internalization of DoxyNPs after 48 h of incubation (Figure S2). Super-resolution microscopy images were acquired to elucidate the intracellular trafficking and subcellular localization of DoxyNPs in MDA-MB-231 cells. Figure 4 shows the multicolor STORM images of the MDA-MB-231 cells exposed to DoxyNPs (red signal) for 24 h (Figure 4A,B and Figure S3) and 48 h (Figure 4C,D and Figure S3), fixed and stained for early and late endosomes/lysosomes (green signal). Subcellular localization of DoxyNPs (Figure 4E,F) was determined by analyzing the images of 15 treated cells and counting the DoxyNPs that were non-colocalized (red signals) and colocalized (red/green signals) with the endolysosomes.

After 24 h of exposure to MDA-MB-231, approximately 60% and 40% of DoxyNPs were found to be colocalized with early and late endosomes, respectively (Figure 4E,F), and the extent of colocalization with both organelles decreased over a longer incubation time. Compared with 24 h of incubation, the colocalization with early endosomes decreased by 20%, and that with late endosomes also decreased by 10% after 48 h of incubation. The reduction of colocalization between particles and compartments indicated the endosomal escape of DoxyNPs after 48 h of incubation. This indicates that DoxyNPs can reach their target sites in the cytosol and exert their desired effects within cells. 

Previous studies showed that most endosomal escape of nanoparticles could be attributed to the “proton sponge effect” [21,27,33,34]. The buffering effect of nanoparticles at pH 5.5~6.5 causes first the diffusion of protons (H^+^) in the endosomes and then the destabilization of the endosomal membrane by osmotic effects. A potentiometric titration was performed to gain more insights into the buffering capacity of DoxyNPs and the mechanism of endosomal escape of the nanodrug (Figure S4). The titration curve of DoxyNPs (Figure S3) did not exhibit obvious buffering capacity in the pH range of 5.5–6.5, which means that the endosomal escape of intact DoxyNPs probably cannot be ascribed to the “proton sponge effects”. Since the DoxyNPs can slowly disassemble at pH 5 (Figure 2A), we hypothesized that released drug molecules could cross the endo-lysosomal membrane and access the cytosol by diffusion [21]. Overall, our results suggested that the endocytic trafficking of DoxyNPs occurs through early and late endosomes during the first 24 h, and then upon disassembly, the drug molecules are released in the cytosol.

**Figure 5:**
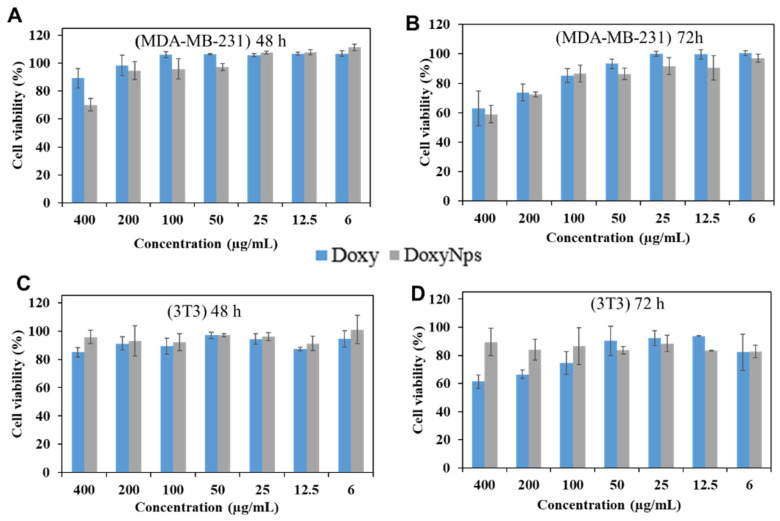
Cytotoxicity tests (MTT assay) of DoxyNPs on MDA-MB-231 cells after (**A**) 48 h and (**B**) 72 h incubation at different concentrations; Cytotoxicity tests (MTT assay) of DoxyNPs cells on fibroblast cells (3T3) after 48 (**C**) and 72 h (**D**) incubation at different concentrations.

We then evaluated the cytotoxicity of modified DoxyNPs after 48 h and 72 h of incubation (Figure 5A,B). Breast cancer cells (MDA-MB-231) were treated with Doxy and DoxyNPs at different concentrations, respectively, and analyzed by the MTT (3-(4,5-dimethylthiazol-2-yl)-2,5-diphenyltetrazolium bromide) assay. It is noted that doxycycline and DoxyNPs possess similar cytotoxicity in MDA-MB-231 cells at prolonged incubation times (72 h). In particular, Doxy and DoxyNPs showed 62 ± 11% and 59 ± 6% cell viability, respectively, at 400 µg/mL (Figure 5B). We can therefore infer that within experimental error limits, the two systems exhibit a similar IC_50_. Overall, these results suggest that DoxyNPs maintain the ability to suppress cancer cell growth. To gain more insight into the cytotoxicity mechanism of DoxyNPs, the normal cell line fibroblast (3T3) was also treated with DoxyNPs. Interestingly, it was found that doxycycline and DoxyNPs have limited antiproliferative activity on fibroblast cells after 48 h of incubation (Figure 5C). With increasing incubation time (Figure 5D), DoxyNPs still maintained high cell viability (89%), whereas unmodified doxycycline only showed 60% cell viability. Overall, these results suggested that the original Doxy molecules exerted toxicity against both cancer and healthy cells; in contrast, DoxyNPs showed selective antiproliferative activity on cancer cells and limited toxicity on healthy fibroblasts (Figure S5).

**Figure 6:**
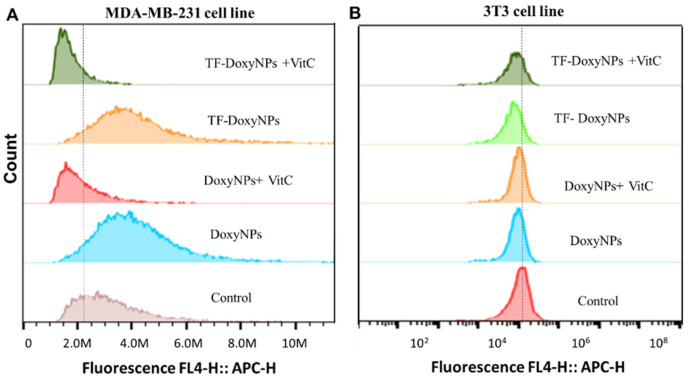
Flow cytometry assay showing ROS (reactive oxygen species) generation in MDA-MB-231 cells (**A**) and 3T3 cells (**B**) treated with DoxyNPs (200 μg/mL) in the presence or absence of radical scavengers (Vitamin C) for 24 h. Transferrin-coated nanoparticles did not affect ROS (reactive oxygen species) generation.

Next, we investigated the mechanism of action underlying the anticancer effect and selectivity of DoxyNps. It has been reported that doxycycline can induce mitochondrial dysfunction and oxidative damage in cancer cells through intracellular ROS generation [29]. We hypothesized that DoxyNPs toxicity is likely mediated by ROS (reactive oxygen species) generation in MDA-MB-231 cells. Hence, flow cytometry was used to assess the ROS production ability of DoxyNPs. It was found that the fluorescence intensity peak was shifted to the right after doxycycline treatment, which means the ROS level in the treated MDA-MB-231 cells was higher than that in the untreated cells (Figure 6A). To confirm that doxycycline acts on breast cancer cells via oxidative damage, we investigated whether antioxidant vitamin C can reduce the effects of doxycycline in breast cancer cells. In the presence of the radical scavenger vitamin C, it was found that the effect of doxycycline on inducing oxidative stress was abolished by vitamin C in MDA-MB-231 cells. Importantly, the left shift of the fluorescence intensity peak to its original level in untreated cells suggests ROS generation is essential for the action of DoxyNPs in MDA-MB-231 cells. 

On the other hand, it was found that there is limited ROS production in healthy fibroblast cells (3T3) after treatment with DoxyNPs (Figure 6B). The different levels of ROS production in cancer cells and healthy cells may explain the difference in cell viability (Figure 5). In general, cancer cells have an enhanced ability to generate and use reactive oxygen species (ROS) compared to healthy cells [35,36]. Although ROS plays a crucial role in normal cellular processes, cancer cells exploit increased ROS signaling to promote tumor growth and progression [27]. However, due to their elevated baseline ROS levels, neoplastic cells are more susceptible to sustained oxidative stress, leading to cell death [37]. Interestingly, these results show that the internalized DoxyNPs exert a limited radical scavenging activity while primarily compromising mitochondrial activity, which results in enhanced ROS production. Overall, our results (Figure 6A,B) indicate that the internalized DoxyNPs selectively exert ROS-mediated cytotoxicity on breast cancer cells, with limited cytotoxicity on healthy cell lines. 

## 3. Materials and Methods

### 3.1. Materials

Doxycycline monohydrate, apo-Transferrin human, bovine serum albumin (BSA), sodium bicarbonate (NaHCO_3_), sodium hydroxide (NaOH), and hydrochloric acid (HCl) were purchased from Sigma-Aldrich (St. Louis, MO, USA). Fluorescence probes NHS Ester Alexa Fluro 488, 556, and 647 were obtained from Invitrogen™. MDA-MB-231 (ATCC HTB-26) and 3T3 (ATCC^®^ CRL-1658™)) cell lines were cultured in Dulbecco’s modified Eagle medium (DMEM) with 10% FBS. Rabbit anti-early endosome antigen 1 (EEA-1) monoclonal antibodies and rabbit anti-Rab7 monoclonal antibodies were supplied by Cell Signaling Technology. The goat anti-rabbit secondary antibody Alexa Fluor 647 was purchased by Invitrogen.

### 3.2. Sonochemical Synthesis of DoxyNPs

Doxycycline nanoparticles (DoxyNPs) were synthesized using an ELAC Nautik generator and an ultrasonic transducer (T&C Power Conversion, Inc.) operating at 490 kHz. To prepare a 1 mg/mL doxycycline solution, the doxycycline powder was heated in a suspension at 55 °C with a magnetic stirrer (600 rpm) for 15 min until fully dissolved. The doxycycline solution (5 mL) was then placed in a sealed glass vial in a custom-made glass cell with a water jacket connected to a water bath to maintain a constant temperature of 37 °C ± 2 °C. Sonication at 14.4 W cm^−3^ measured by calorimetry was applied to initiate the sono-assembly reaction for 1 h. The purified nanoparticles were obtained by washing with Milli-Q water three times (centrifuge at 7000 rpm for 10 min). Following our previous studies, the composition of DoxyNps has been analyzed by mass spectrometry, NMR, and HPLC. The size and morphologies of DoxyNPs were observed using a scanning electron microscope (FEI Teneo Volume Scope) at an acceleration voltage of 10 keV. DoxyNPs were labeled using the NHS ester Alexa Fluro 647 for stochastic optical reconstruction microscopy (STORM) imaging.

### 3.3. Protein Adsorption and Bio-Stability Test

To assess the biostability of DoxyNPs, particles were incubated with 10% Fetal bovine serum (FBS) and 100% FBS medium for 3 h, respectively. The size and zeta potential of particles were measured by dynamic light scattering (DLS) with a Zetasizer (Malvern Instruments Ltd, Malvern, UK). The release kinetics of DoxyNPs were investigated by incubating the particles (1 mg/mL) in four different aqueous mediums: pH~5 and pH~7.4 PBS buffer (100 mM), cell culture medium containing 10% FBS (Dulbecco’s Modified Eagle Medium (DMEM) + 10% FBS), and 100% FBS at 37 °C under stirring/shaking. The suspension was centrifuged (7000 *g*, 8 min) at different time points, and then the supernatant was collected for analysis. The supernatant was measured by fluorescence spectroscopy at an excitation wavelength of 360 nm. The intensity of the fluorescence emission spectrum was used to calculate the dissolution percentage of DoxyNPs. For STORM (Stochastic Optical Reconstruction Microscopy) analysis, DoxyNPs were first labeled with Alexa Fluro NHS-Ester 647, then incubated with different concentrations of FBS medium. After 3 h, one drop of particle suspension was added to the circular glass slide coated with positive-charged polyethylenimine (1 mg/mL PEI + 0.5 M NaCl). Then sufficient imaging buffer was added on top of the sample suspension for imaging acquisition. The imaging buffer can be prepared as follows: (i) two stock buffers: buffer A (10 mM Tris, pH~8 + 50 mM NaCl) and buffer B (50 mM Tris, pH~8 +10 Mm NaCl, +10% glucose); (ii) GLOX solution (250 µL): 14 mg glucose oxidase +50 µL catalase (17 mg/mL) +200 µL buffer A; (iii) 1 mol/L MEA (1 mL): Cysteamine hydrochloride (0.113 g + 1 mL Milli-Q); (iv) Imaging buffer: on ice, 7 µL GLOX and 70 µL 1 M MEA were added to 620 µL buffer B and vortex gently to mix for use.

### 3.4. Association of DoxyNPs by MDA-MB 231 Cells

The association and uptake of DoxyNPs by MDA-MB-231 (breast cancer) cells were evaluated using flow cytometry. DoxyNPs were coated with transferrin dual-labeled by 556 and 647 (ratio 1:2:0.6) Alexa Fluor NHS-Ester in 1 M NaHCO_3_. DoxyNPs (100 µg/mL) were incubated with cells for 2 h, 4 h, 19 h, and 24 h in a 24-well plate (Costar 3596, Corning, MA, USA). The cells were detached with trypsin (200 µL 5%), and then samples were harvested, washed twice with 500 µL 1% BSA/PBS, and centrifuged at 400 rpm for 5 min at 4 °C. The washed cell pellet was resuspended in 1% BSA/PBS for analysis. The cell suspensions were analyzed by a flow cytometer by selecting appropriate FSC vs. SSC gates to exclude debris and cell aggregates under FL4 channels, and the software "FlowJo" was used for data analysis.

### 3.5. Determination of Cytotoxicity of Nanoparticles

The cytotoxicity of the Doxy NPs was determined using the MTT [3-(4,5-dimethylthiazol-2-yl)-2,5-diphenyl-2H-tetrazolium bromide)] assay. MDA-MB-231 and fibroblast cells (ATCC^®^ HTB-26TM, Manassas, VA, USA) were plated on 96-well plates (Costar 3596, Corning, MA, USA) with a desired seeding density in 100 µL of DMEM medium supplemented with 10% fetal bovine serum (FBS) and incubated at 37 °C, 5% CO_2_, for 24 h for cells to attach. Then, 1 mg/mL Doxy NPs were prepared and diluted accordingly. Afterwards, DoxyNPs were added to the culture media and incubated for up to 72 h at a final concentration of 400, 200, 100, 50, 25, 12.5, 6, and 3 µg/mL respectively. Cell viability was determined by measuring the well absorbance at 554 nm and 670 nm as a reference with an Infinite M200 microplate reader (Tecan, Zurich, Switzerland). For comparison, the cytotoxicity of standard doxycycline was also studied following the same procedure as the MTT assay.

### 3.6. Detection of Reactive Oxygen Species

ROS production was determined in MDA-MB-231 cells using the Cellular Reactive Oxygen Species Detection Assay Kit (Deep Red Fluorescence) (AbCam, Melbourne, Australia). MDA-MB-231 cells (10,000 per well) were plated into 96-well plates for 24 h and then incubated with 200 μg/mL DoxyNPs in the presence or absence of radical scavenger vitamin C (100 μM). After 24 h of incubation under low-light conditions, a ROS-specific (deep red) detection probe was added to cells and incubated for 60 min in a 37 C/5% incubator. The cells were then harvested (using the same method as mentioned in Section 3.4) and immediately analyzed by flow cytometry at the FL4 channel. 

### 3.7. Intracellular Trafficking Study by STORM

DoxyNPs were coated with transferrin dual-labeled by 556 and 647 (ratio 2:1) Alexa Fluor NHS-Ester in 1 M NaHCO_3_. MDA-MB-231 cells were seeded at a seed density of 40,000 cells per well in NuncTM Labtek 8-well chamber coverglass slides (Thermo Fisher Scientific, Scoresby, Australia) and incubated overnight (37 °C and 5% CO_2_) for cell attachment. To monitor the interaction pathway between DoxyNPs and cells, 4 coverglass slides with attached cells were prepared by refreshing medium and adding dual-labeled DoxyNPs (556/647) to a final concentration of 200 µg/mL. After 24 h of incubation, the medium was removed from one of the wells, and cells were washed three times with PBS to remove unbound particles. In the remaining wells, the medium was refreshed for further incubation until 48 h, respectively. 

The washed cells were fixed with 4% paraformaldehyde (200 µL/well) for 15 min at room temperature and washed three times with PBS. Cells were then permeabilized for 5 min using a 0.1% Triton X-100 solution in PBS (200 µL/well) and washed three times with PBS. To minimize non-specific interactions and false-positive staining, 2.5% PBS-BSA was used to block samples for 1 h in the dark. Afterwards, blocking solution was removed, and different primary antibodies were added to samples, namely rabbit anti-EEA1 monoclonal antibody and rabbit anti-Rab7 monoclonal antibody for the early endosomes and late endosomes/lysosomes (5 µg/mL) (dilution 1:200, using 1% BSA for dilution), respectively. After 2 h of incubation in the dark, cells were washed three times with PBS and incubated for 1.5 h with 5 µg/mL goat anti-rabbit (for early and late endosome) IgG secondary antibody dual-labeled by Alexa Fluor NHS-Ester 488/647. The dual-labeled secondary antibody was primarily prepared by mixing activator dye (488) and reporter dye (647) in 1 M NaHCO_3_, following the mole ratio of 2:1. The mixtures covered by aluminum foil were incubated for 30 min on a shaking platform. After incubation, samples were passed through the NAP-5 gel filtration column to get rid of excess dye.

The washed samples can be stored in the fridge for further analysis. To gain insight into subcellar structures, multicolor STORM was performed by the activator (488 or 556 nm)-reporter (647 nm) dye pairs. 

## 4. Conclusions

In this work, we have introduced a simple ultrasonic technology for transforming the physicochemical structure and functionality of an existing antibiotic, providing an alternative and innovative approach for the discovery of new carrier-free nanodrugs. Doxycycline has been successfully transformed into a selective carrier-free anticancer nanodrug by using an innovative ultrasound-assisted self-assembling strategy. The DoxyNPs were found to be stable in mimicking physiological conditions and capable of gradually releasing the drug over a long period of time. Compared with unmodified doxycycline, DoxyNPs showed selective toxicity against cancer cells while showing limited effects on healthy cells due to the different levels of ROS generation in cells. These findings open possibilities for using DoxyNPs in cancer therapy and reducing side effects as well. Unlike small drug molecules that are rapidly metabolized and excreted by the body, DoxyNPs are entirely composed of drug molecules and could potentially carry a higher dose of active agents and enable the slow release of the therapeutic agent. The fabrication of such carrier-free nanodrugs is also a green process as it does not require organic solvents, initiators, crosslinking agents, or surfactants. Overall, the study suggests that sonochemically synthesized DoxyNPs could be promising carrier-free nanodrugs with potential therapeutic applications.

## Data Availability

The data presented in this study are available on request from the corresponding author.

## Supplementary Materials

The following supporting information can be downloaded at: https://www.mdpi.com/article/10.3390/molecules28134927/s1, Figure S1. Size distribution of DoxyNPs incubated in 10% and 100% FBS for 3 h (A) and 24 h (B) measured by dynamic light scattering after repeated washing. Figure S2. Confocal microscopy imaging of monitoring intracellular interaction between DoxyNPs and MDA-MB-231 cells as function of time up to 24 h. Representative of merged channel images of the MDA-MB-231 cells exposed for 3 h, 5.5 h, 8 h, 24h to DoxyNPs (red signal) and stained for early and late endosome (green signal). Blue signal represents nuclei acid, stained with Hoechst. Figure S3. Intracellular tracking study of DoxyNPs in MDA-MB-231 cells by stochastic optical reconstruction microscopy (STORM). Representative multicolour STORM images of the MDA-MB-231 cells exposed for 24 h to DoxyNPs (red signal) and stained for early (A) and late endosome (B) (green signal). Figure C and D represent that of 48 h for early and late endosome, respectively. Scale bar is 10 µm. Figure S4. A potentiometric titration was performed to gain more insights into the mechanism of cellular interaction with DoxyNPs. The titration curve of DoxyNPs represented changes in pH as function of OH- concentration (addition of 0.01 M NaOH) in solution. DoxyNPs did not exhibit obvious buffering capacity in pH range of 5.5-6.5, which means that the endosomal escape of DoxyNPs probably cannot be ascribed to the “proton sponge effects”. Figure S5. Cytotoxicity tests (MTT assay) of DoxyNPs on MDA-MB-231cells and Fibroblast (3T3) after 72 h incubation at different concentrations. DoxyNPs showed selective antiproliferative activity on cancer cells and limited toxicity on healthy fibroblasts.
