# Supplementary material for: Ultrasonic Transformation of Antibiotic Molecules into a Selective Chemotherapeutic Nanodrug
Source: Molecules. 2023 Jun 22;28(13):4927. doi: 10.3390/molecules28134927 (PMC10343459; doi:10.3390/molecules28134927)
Supplement: Supplementary file 1 [file molecules-28-04927-s001.zip › molecules-2434770-supplementary.pdf]

Supplementary Materials

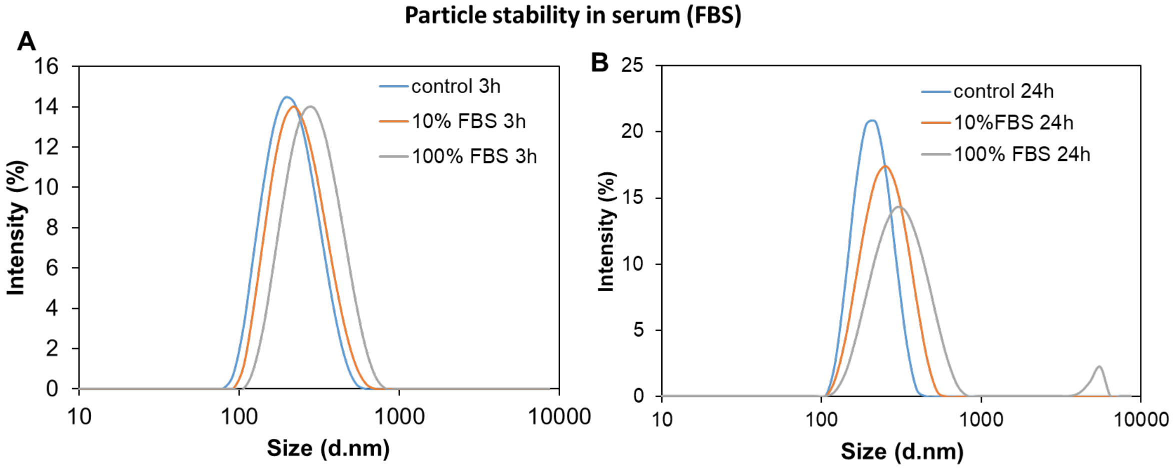

Figure S1. Size distribution of DoxyNPs incubated in 10% and 100% FBS for 3 h (A) and 24 h (B) measured by dynamic light scattering after repeated washing.

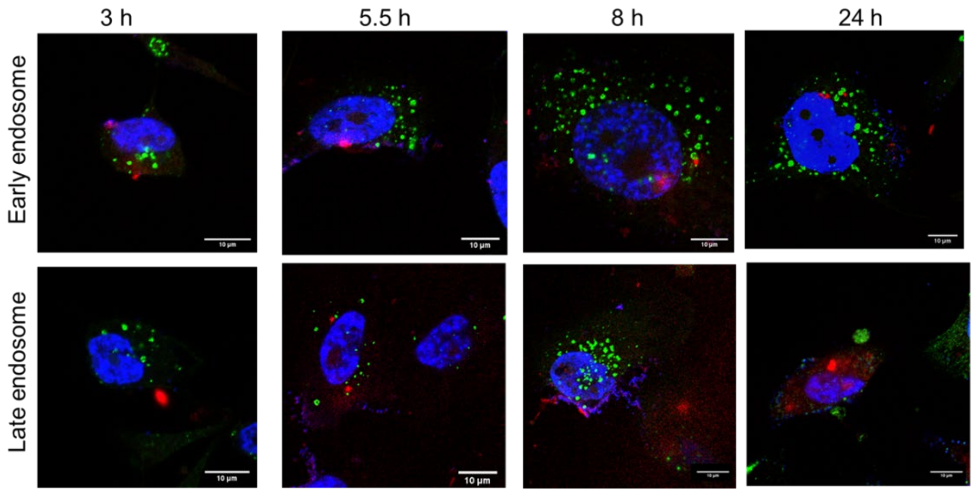

Figure S2. Confocal microscopy imaging of monitoring intracellular interaction between DoxyNPs and MDA-MB-231 cells as function of time up to 24 h. Representative of merged channel images of the MDA-MB-231 cells exposed for 3 h, 5.5 h, 8 h, 24h to DoxyNPs (red signal) and stained for early and late endosome (green signal). Blue signal represents nuclei acid, stained with Hoechst.

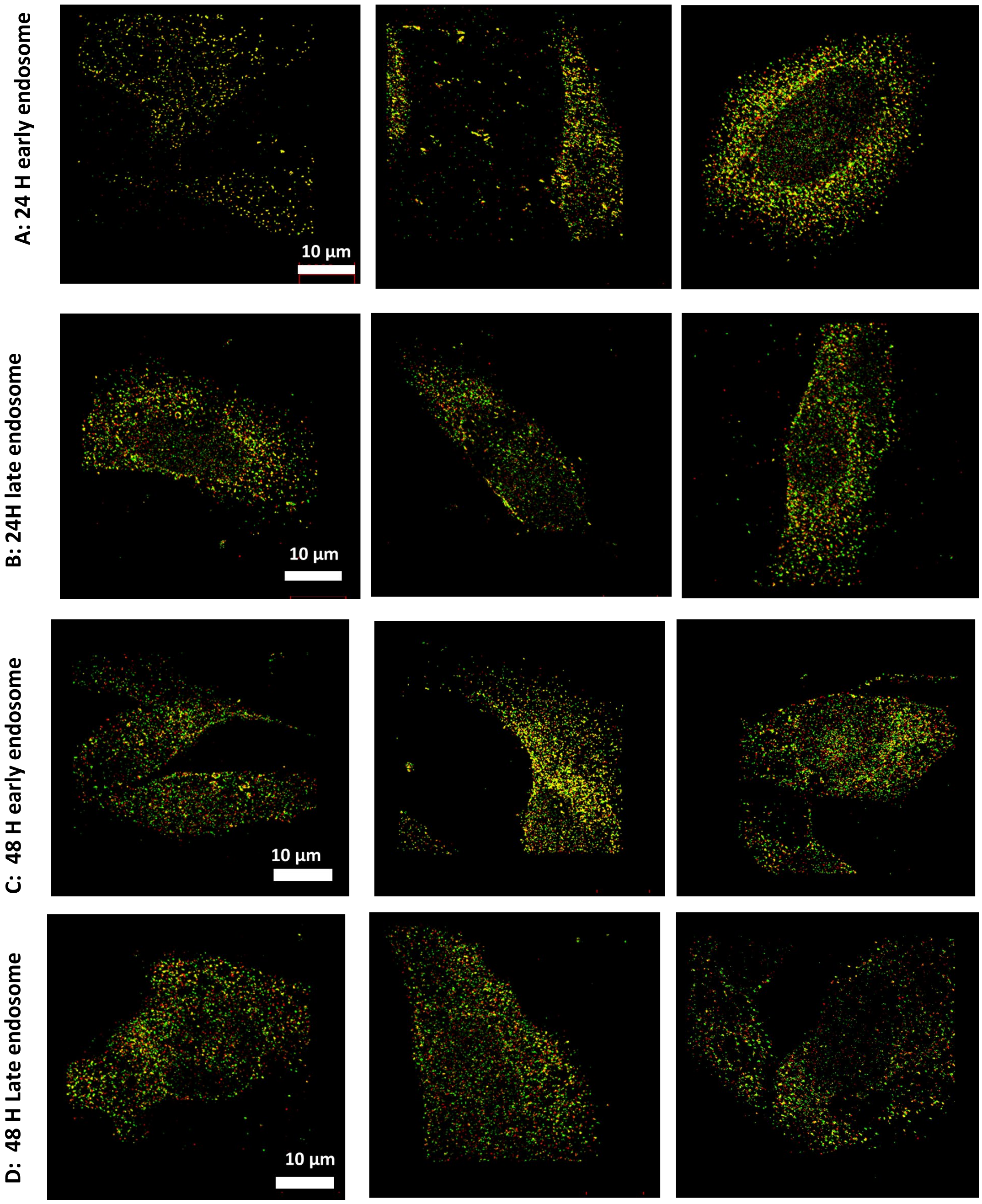

Figure S3. Intracellular tracking study of DoxyNPs in MDA-MB-231 cells by stochastic optical reconstruction microscopy (STORM). Representative multicolour STORM images of the MDA-MB-231 cells exposed for 24 h to DoxyNPs (red signal) and stained for early (A) and late endosome (B) (green signal). Figure C and D represent that of 48 h for early and late endosome, respectively. Scale bar is 10 µm.

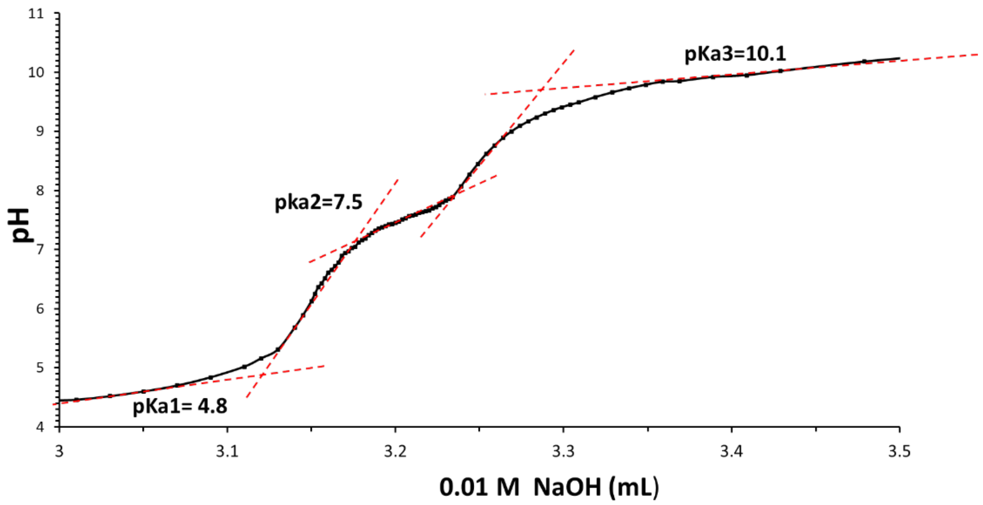

Figure S4. A potentiometric titration was performed to gain more insights into the mechanism of cellular interaction with DoxyNPs. The titration curve of DoxyNPs represented changes in pH as function of OH- concentration (addition of 0.01 M NaOH) in solution. DoxyNPs did not exhibit obvious buffering capacity in pH range of 5.5-6.5, which means that the endosomal escape of DoxyNPs probably cannot be ascribed to the "proton sponge effects".

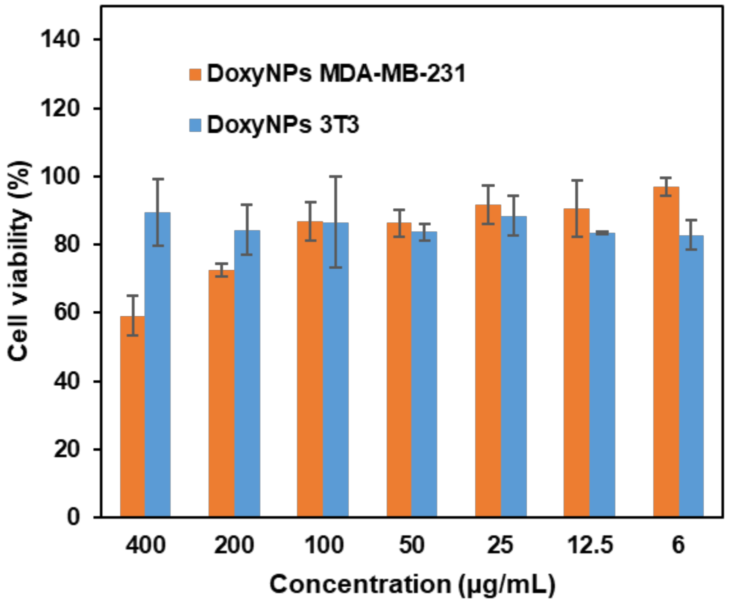

Figure S5. Cytotoxicity tests (MTT assay) of DoxyNPs on MDA-MB-231cells and Fibroblast (3T3) after 72 h incubation at different concentrations. DoxyNPs showed selective antiproliferative activity on cancer cells and limited toxicity on healthy fibroblasts.
